# Glycated haemoglobin as a predictor for metabolic syndrome in non-diabetic Korean adults

**DOI:** 10.1111/j.1464-5491.2007.02146.x

**Published:** 2007-08-01

**Authors:** K C Sung, E J Rhee

**Affiliations:** Department of Internal Medicine, Kangbuk Samsung Hospital, Sungkyunkwan University School of Medicine Seoul, Korea

**Keywords:** fasting hyperglycaemia, glycated haemoglobin, metabolic syndrome

## Abstract

**Aims:**

With increasing prevalence of diabetes mellitus and metabolic syndrome (MS), the importance of early detection of insulin resistance is emphasized. However, a simple and practical method of measurement is not readily available. Therefore, we examined the sensitivity and specificity of HbA_1c_ for predicting impaired fasting glucose (IFG) and MS and its association with cardiovascular risk factors, particularly in the normal range of HbA_1c_ levels in non-diabetic Korean subjects.

**Methods:**

In 40 155 participants (median age 40 years) participating in a medical check-up programme, analysis of the distribution of HbA_1c_ and its association with various cardiovascular risk factors was performed. In 22 465 selected participants, an analysis was conducted of the ability of HbA_1c_ to predict MS and IFG. Anthropometric measurements were made in all subjects and fasting glucose, lipid profiles and HbA_1c_ were measured. The presence of MS was defined according to the definitions of the Adult Treatment Panel III (ATP III) guideline and the new International Diabetes Federation (IDF) guideline. Patients with diabetes were excluded from the study.

**Results:**

The incidence of MS was 12.2% according to ATP III criteria and 7.6% according to IDF criteria. When subjects were grouped by quartile of HbA_1c_, cardiovascular risk factors significantly increased as the HbA_1c_ increased. An HbA_1c_ of 5.45% predicted the presence of MS (ATP III: sensitivity/specificity 57.4/64.3%, area under the curve 64.8%; IDF: sensitivity 60.2/63.4%, area under the curve 66.1%) and fasting blood glucose ≥ 5.6 mmol/l (sensitivity/specificity 53.7/70%, area under the curve 66.1%). When the analyses were done separately by gender, female subjects showed higher cut-off of HbA_1c_ for the prediction of MS (5.55% for both ATP III and IDF criteria).

**Conclusions:**

HbA_1c_ increased as cardiovascular risk factors increased and HbA_1c_ of 5.45% predicted the presence of MS. HbA_1c_ might be a predictive measure of IFG and MS, and also cardiovascular risk factors in the Korean population.

Diabet. Med. 24, 848–854 (2007)

## Introduction

Diabetes mellitus affects 5% of the world's population and its prevalence is doubling every generation [1]. Between 1996 and 2005, the number of people diagnosed with diabetes in the UK increased from 1.4 million to 2.1 million, including 1.8 million diagnosed with Type 2 diabetes. This number has increased by around 50% in < 10 years [2]. Diabetes is associated with many cardiovascular risk factors, which may be present before the onset of hyperglycaemia or develop after the diagnosis of diabetes [3,4]. The metabolic syndrome (MS) is a cluster of various diseases, such as hypertension, obesity, dyslipidaemia and hyperglycaemia, in which insulin resistance plays a key pathogenic role, and it has been proposed that this syndrome is a powerful determinant of diabetes and cardiovascular disease (CVD) [5–7].

Evidence is accumulating that macrovascular disease is associated with lesser degrees of hyperglycaemia than microvascular disease [8,9]. The heightened risk of CVD extends to impaired glucose tolerance (IGT) and, as with diabetes, IGT is often associated with MS, the components of which explain some, but not all of the excessive CVD risk seen in IGT and diabetes [10]. Both IGT and impaired fasting glucose (IFG) are very strong risk markers for the development of diabetes and recent reports support the importance of both categories as risk factors of CVD [11].

Glycated haemoglobin (HbA_1c_) is widely accepted as a useful index of mean blood glucose and therapeutic guideline of diabetes. HbA_1c_ may predict incident cardiovascular events, even in individuals without diabetes mellitus [12–14], although Blake *et al*. have recently reported that the association of HbA_1c_ with future cardiovascular risk in women without diabetes mellitus is largely attributable to a strong correlation with other cardiovascular risk factors. Thus other proatherogenic effects of diabetes, rather than levels of glycaemia, might be related to the vascular complications of diabetes [15]. Recent work suggests the utility of HbA_1c_ as the predictor of future risk for diabetes mellitus in diverse ethnic groups [16].

Although there is no doubt that insulin resistance is the major aetiological factor in the development of MS, direct quantitative measurement of insulin sensitivity is not readily available and thus cannot be used as the diagnostic tool for the syndrome. Thus, various diagnostic criteria for MS have been suggested [17]. A recent study by Osei *et al*. [18] examined the significance of HbA_1c_ as a surrogate for MS in high-risk African-Americans who were genetically predisposed to Type 2 diabetes. They demonstrated that in subjects with increased HbA_1c_, some, but not all, components of MS could be defined by HbA_1c_. No further research has investigated cut-offs of HbA_1c_ in the diagnosis of MS. Therefore, we examined the association of HbA_1c_ with the components of MS and attempted to determine cut-offs of HbA_1c_ in the diagnosis of MS in a large non-diabetic Korean population. Our aim was to determine whether HbA_1c_ could be used as a simple method to select those at risk of MS.

## Patients and methods

### Patients

From participants in the annual medical check-up programme of the Health Promotion Centre in Kangbuk Samsung Hospital, Sungkyunkwan University School of Medicine from January to December 2005, 40 155 subjects were enrolled. Patients with diabetes mellitus were specifically excluded from the study and those suspected of having acute inflammatory disease, malignancies and those taking lipid-lowering medication, antihypertensive agents or glucose-lowering agents were excluded; 40 155 subjects were included in analysis of the distribution of HbA_1c_ and its association with various cardiovascular risk factors. Furthermore, we selected 22 465 subjects whose waist circumference data were available for the definition of MS. We conducted the analysis for the cut-offs for the diagnosis of MS and IFG.

Data related to smoking, alcohol consumption and exercise were obtained from self-completed surveys at the time of the health check-up. Subjects were divided into three groups according to smoking status: non-smoking, past smoking and current smoking groups; and into four groups according to drinking status: non-drinking, 3–4 times per month, 1–2 times per week, 3–4 times per week and > 5 times per week. Subjects were divided into three groups according to exercise status: no exercise at all, less than 3 times a week and more than 3 times a week. The data were collected from the notes review of the participants and the protocol was reviewed by the Institutional Review Board of Kangbuk Samsung Hospital.

### Anthropometric measurements and blood sampling

Height, weight, waist circumference, and systolic and diastolic blood pressure were measured in duplicate and the results averaged. Blood pressure was measured with a standardized sphygmomanometer after at least 5 min of rest, according to the Hypertension Detection and Follow-up Program protocol. Body mass index (BMI) was calculated by dividing weight (kg) by height (m) squared.

After a 12-h fast, blood glucose, total cholesterol, triglyceride, high-density lipoprotein cholesterol (HDL-C) and low-density lipoprotein-cholesterol (LDL-C) levels were measured. The hexokinase method (Advia 1650 Autoanalyser; Bayer Diagnostics, Leverkusen, Germany) was used to measure blood glucose levels and an enzymatic colorimetric test was used to measure total cholesterol and triglyceride levels. The selective inhibition method was used to measure HDL-C and a homogeneous enzymatic colorimetric test was used to measure LDL-C. Apoprotein B and Apoprotein A1 were measured on a BN II system (Dade Behring Co., Marburg, Germany).

HbA_1c_ was measured by immunoturbidimetric assay with a Cobra Integra 800 automatic analyser (Roche Diagnostics, Basel, Switzerland) with a reference value of 4.4–6.4%. The methodology was aligned with the Diabetes Control and Complications Trial (DCCT) and National Glycohemoglobin Standardization Program (NGSP) standards [19]. The intra-assay coefficient of variation (CV) was 2.3% and interassay CV was 2.4%, both within the NGSP acceptable limits [20].

Serum insulin concentration were measured with an immunoradiometric assay (INS-Irma; Biosource, Nivelles, Belgium), with intra- and interassay CVs of 1.6–2.2% (mean serum concentration 6.6 ± 0.1, 53.3 ± 0.8 µIU/ml) and 6.1–6.5% (mean serum concentration 14.4 ± 0.9, 100.4 ± 6.1 µIU/ml), respectively. As a marker of insulin resistance, homeostatic model assessment (HOMA)-insulin resistance (IR) was calculated as follows [21]: HOMA-IR =[fasting insulin (µIU/ml) × fasting glycaemia (mmol/l)]/22.5.

### Diagnosis of IFG and MS

IFG was defined according to newly recommended criteria by the American Diabetes Association: fasting glucose ≥ 5.6 mmol/l [22].

We applied two different definitions of MS in this population: the minor modification version of Third Report National Cholesterol Education Program Expert Panel on Detection, Evaluation, and Treatment of High Blood Cholesterol in Adults (NCEP-ATP III) guidelines [23] and the newly recommended guideline by the International Diabetes Federation (IDF) [24].

The NCEP-ATP III definition is satisfied when more than three of the following five criteria are met:

1. Waist circumference: ≥ 90 cm in men, ≥ 80 cm in women (defined by the Western Pacific Region of WHO for obesity (WPRO) criteria [25]).
2. Hypertriglyceridaemia: ≥ 1.7 mmol/l.
3. Low HDL-C: < 1.0 mmol/l in males and < 1.3 mmol/l in females.
4. Hypertension: ≥ 130/85 mmHg.
5. Fasting hyperglycaemia: ≥ 5.6 mmol/l.

According to the new IDF definition, for a person to be defined as having MS they must have [24] central obesity defined with ethinicity-specific values (≥ 90 cm in men, ≥ 80 cm in women) plus any two of the following four factors:

1. Hypertriglyceridaemia: ≥ 1.7 mmol/l, or specific treatment for this lipid abnormality.
2. Low HDL-C: < 1.0 mmol/l in males and < 1.3 mmol/l in females, or specific treatment for this lipid abnormality.
3. Hypertension: ≥ 130/85 mmHg, or treatment of previously diagnosed hypertension.
4. Fasting hyperglycaemia: ≥ 5.6 mmol/l.

### Statistical methods

Results are expressed as median and range, and since no variables assessed in the study were normally distributed. Non-parametric tests were used to perform the analysis. The Kormogorov–Smirnov test was used to test normality. Comparisons of parameters between groups were made using Mann–Whitney *U*-test and Kruskal–Wallis *H*-test, and comparisons after adjustment for confounding factors were performed using ancova test. In this study, receiver–operating characteristic (ROC) curves for predicting IFG and MS were derived by plotting the sensitivity vs. 1—specificity. The optimal cut-off point was defined as the closest point on the ROC curve to the point where 1—specificity was 0 and sensitivity was 100%. The areas under the curve represent the probability that a subject chosen at random, who had IFG or MS, had a higher test value than a subject who did not have IFG or MS. The level of significance was chosen as *P* < 0.05. All analyses were carried out with the statistical program SPSS for Windows v. 12.0 (SPSS Inc., Chicago, IL, USA).

## Results

Of the 40 155 subjects, 24 921 (62.1%) were males and 15 234 (37.9%) females and median age was 40 years (Table 1). Mean concentration of HbA_1c_ was 5.32% in men and 5.41% in women. Of 22 465 subjects whose waist circumference values were available, 12.2% satisfied the diagnostic criteria for MS by ATP III criteria and 7.6% by IDF criteria (Table 1).

**Table 1:** General characteristics of the study participants

*N* = 40 155	Median (range)
Age (years)	40.1 (20–86)
Male (%)	24 921 (62.1)
Fasting blood glucose (mmol/l)	5.2 (3.16–6.94)
HbA_1c_ (%)	5.4 (3.5–7.6)
Total cholesterol (mmol/l)	4.9 (2.17–11.72)
HDL-C (mmol/l)	1.3 (0.52–3.88)
LDL-C (mmol/l)	2.1 (0.01–8.79)
Triglyceride (mmol/l)	2.8 (0.65–55.47)
Uric acid (µmol/l)	321.2 (23.79–713.76)
Hs-CRP (mg/dl)	0.05 (0.02–12.00)
Fasting insulin (µIU/ml)	8.1 (2.08–73.17)
HOMA-IR	1.9 (0.40–18.59)
Apolipoprotein A1 (g/l)	1.4 (0.20–4.88)
Apoliproprotein B (g/l)	0.9 (0.24–2.59)
Systolic blood pressure (mmHg)	110 (70–210)
Diastolic blood pressure (mmHg)	76 (40–130)
Body mass index (kg/m^2^)	23.4 (14.7–44.2)
Waist circumference (cm)	79.0 (52–136)
Metabolic syndrome (%, *N* = 22 465)*	
By ATP III criteria	2730 (12.2)
By IDF criteria	1704 (7.6)

HDL-C, High-density lipoprotein cholesterol; LDL-C, low-density lipoprotein cholesterol; hs-CRP, high-sensitivity C-reactive protein; HOMA-IR, homeostasis model assessment-insulin resistance.

* The prevalence of metabolic syndrome was analysed only in subjects whose waist circumference was available.

The mean HbA_1c_ increased significantly with increasing age and BMI (Table 2). Non-smokers had higher mean HbA_1c_ compared with past or current smokers and HbA_1c_ was higher in those who did not drink alcohol compared with those who did (Table 2). Similarly, subjects who exercised regularly at least three times a week had higher mean HbA_1c_ compared with those who did not (Table 2).

**Table 2:** Distribution of HbA_1c_ in non-diabetic subjects by life-style pattern

		Number	Median (range)	*P*-value
By gender	Male	24 921	5.3 (3.5–11.0)	< 0.01
	Female	15 234	5.4 (3.6–8.6)	
	Total	40 155		
By age groups (years)	20–29	734	5.2 (4.0–6.1)	< 0.01
	30–39	19 094	5.3 (3.5–7.0)	
	40–49	15 789	5.4 (3.5–8.6)	
	50–59	3297	5.5 (3.6–7.7)	
	60–69	1088	5.6 (3.7–11.0)	
	≥ 70	153	5.6 (4.5–7.1)	
	Total	40 155		
By smoking status	Non-smoking	21 381	5.4 (3.5–11.0)	< 0.01
	Past smoking	6609	5.3 (3.5–8.6)	
	Current smoking	10 854	5.3 (3.5–7.5)	
	Total	38 844		
By drinking habit	Non-drinking	14 085	5.4 (3.5–11.0)	< 0.01
	≤ 3–4 times per month	10 901	5.3 (3.5–7.5)	
	1–2 times per week	10 851	5.3 (3.7–8.1)	
	3–4 times per week	2788	5.3 (3.7–7.4)	
	≥ 5 times per week	578	5.3 (4.2–7.7)	
	Total	39 203		
Exercise	Non-exercise	11 928	5.3 (3.6–7.9)	< 0.01
	< 3 times per week	13 665	5.3 (3.5–8.0)	
	≥ 3 time per week	13 593	5.3 (3.8–11.0)	
	Total	39 186		
By BMI (kg/m^2^)	< 18.5	1483	5.3 (4.2–8.1)	< 0.01
	18.5–22.9	16 663	5.3 (3.5–7.4)	
	23.0–24.9	10 135	5.4 (3.5–11.0)	
	≥ 25.0	11 494	5.4 (3.9–8.6)	
	Total	39 775		

Data are given as median with range and number of patients.

When the subjects were grouped by quartile of HbA_1c_, atherosclerosis risk factors significantly increased as the HbA_1c_ increased and HDL-C and Apo A levels decreased as HbA_1c_ increased (*P* < 0.01) (Table 3). As expected, the largest number of subjects with MS by either definition was in the highest quartile of HbA_1c_ (Table 3). As in Table 2, more subjects in the highest quartile of HbA_1c_ smoked tobacco and drank alcohol compared with those in the lower quartiles (Table 3).

**Table 3:** Comparison of cardiovascular risk factors by quartile of HbA_1c_

	HbA_1c_ quartile				
		
Characteristics	< 5.1 (*n* = 10 938)	5.1–5.3 (*n* = 9668)	5.3–5.5 (*n* = 12 820)	≥ 5.6 (*n* = 6729)	*P*-value
Age (years)	38.0 (20–85)	39.0 (20–81)	40.0 (20–86)	42.0 (21–84)	< 0.01
Male (%)	7740 (70.8)	6133 (63.4)	7424 (57.9)	3624 (53.9)	< 0.01
Fasting blood glucose (mmol/l)	5.1 (3.1–6.9)	5.2 (3.2–6.9)	5.3 (3.8–7.0)	5.5 (2.7–7.0)	< 0.01
TC (mmol/l)	4.7 (2.0–9.3)	4.8 (2.2–10.3)	4.9 (2.0–12.1)	5.1 (2.4–12.2)	< 0.01
TG (mmol/l)	2.6 (0.6–24.6)	2.6 (0.7–55.5)	2.7 (0.5–53.3)	3.1 (0.6–37.6)	< 0.01
HDL-C (mmol/l)	1.3 (0.6–4.0)	1.3 (0.6–3.2)	1.3 (0.6–3.3)	1.3 (0.5–3.4)	< 0.01
LDL-C (mmol/l)	2.1 (0.01–6.83)	2.1 (0.01–7.1)	2.2 (0.01–8.8)	2.3 (0.01–8.5)	< 0.01
Uric acid (µmol/l)	327.1 (29.7–719.7)	321.2 (35.7–701.9)	315.2 (23.8–666.2)	315.2 (41.6–701.9)	< 0.01
Hs-CRP (mg/dl)	0.04 (0.02–9.6)	0.04 (0.02–12.0)	0.05 (0.02–10.2)	0.06 (0.02–4.0)	< 0.01
Fasting insulin (µIU/ml)	7.6 (2.1–35.0)	7.8 (2.1–73.2)	8.0 (2.1–134.0)	8.6 (2.3–48.8)	< 0.01
HOMA-IR	1.7 (0.4–8.6)	1.8 (0.4–18.6)	1.9 (0.5–32.7)	2.1 (0.5–11.8)	< 0.01
Apo A1 (g/l)	1.4 (0.6–3.3)	1.4 (0.2–2.9)	1.39 (0.2–4.9)	1.38 (0.2–3.1)	0.001
Apo B (g/l)	0.9 (0.2–2.1)	0.9 (0.2–2.0)	0.92 (0.2–2.6)	1.0 (0.2–2.0)	< 0.01
SBP (mmHg)	110 (70–210)	110 (74–208)	110 (70–260)	110 (70–240)	< 0.01
DBP (mmHg)	74 (44–130)	70 (40–130)	70 (44–180)	76 (46–130)	< 0.01
BMI (kg/m^2^)	23.1 (14.7–38.2)	23.1 (13.4–37.5)	23.3 (14.7–44.2)	24.1 (15–41.8)	< 0.01
WC (cm)	78 (54–109)	78 (53–115)	79 (52–136)	81 (52–120)	< 0.01
Current smokers (%)*	3236 (30.3)	2672 (28.5)	3309 (26.7)	1637 (25.7)	< 0.01
Current drinkers (%)*	7619 (79.0)	6248 (65.8)	7624 (60.9)	3627 (56.1)	< 0.01
Regular exercise (%)*†	3571 (33.3)	3296 (34.8)	4416 (35.3)	2310 (35.8)	< 0.01
Metabolic syndrome (%)‡					
ATP III criteria	407 (7.3)	465 (8.7)	860 (11.7)	998 (24.0)	< 0.01
IDF criteria	230 (4.1)	264 (4.9)	556 (7.6)	654 (15.7)	< 0.01

HDL-C, High-density lipoprotein cholesterol; LDL-C, low-density lipoprotein cholesterol; hs-CRP, high-sensitivity C-reactive protein; HOMA-IR, homeostasis model assessment-insulin resistance.

Data are given as median with range and number of patients.

* Proportions were analysed in subjects with available data.

† Regular exercise at least three times a week.

‡ The prevalences of metabolic syndrome were analysed only in subjects whose waist circumference values were available.

All significant differences of the continuous variables between HbA_1c_ quartile were consistent even after adjustment for age, BMI and gender by ancova test.

An HbA_1c_ of 5.45% predicted the presence of MS diagnosed according to the ATP III guideline with a sensitivity of 57.4% and specificity of 64.3% (area under the curve 64.8%), of MS according to the IDF guideline with a sensitivity of 60.2% and specificity of 63.4% (area under the curve 66.1%) and fasting hyperglycaemia with a sensitivity of 53.7% and specificity of 70% (area under the curve 66.1%) (Table 4).

**Table 4:** ROC curve of HbA_1c_ to predict impaired fasting glucose and metabolic syndrome by two criteria

	Sensitivity/specificity (%)								
	
Cut-offs (%)	Total			Male			Female		
			
	ATP III	IDF	IFG	ATP III	IDF	IFG	ATP III	IDF	IFG
5.35	68.1/51.2	71.0/50.5	65.2/56.9	64.9/55.9	68.4/54.9	61.7/63.1	79.8/44	78.5/43.5	75.1/48.3
5.45	57.4/64.3	60.2/63.4	53.7/70.0	53.4/68.7	56.5/67.5	49.9/75.8	72.4/57.5	70.9/56.9	64.3/62
5.55	47.5/75.2	49.9/74.3	42.8/80.6	42.6/78.9	45.1/77.5	38.8/85.2	65.2/69.7	63.8/69	53.9/74.1
5.65	36.6/84	38.4/83.1	32.6/88.6	32.1/86.4	33.8/85.3	29.0/91.7	52.9/80.2	51.7/79.6	42.4/84.3
AUC	64.8	66.1	66.1	64.8	66.0	67.3	71.9	70.4	68.9

ROC, Receiver–operator curve; ATP, Adult Treatment Panel; IDF, diagnostic criteria for metabolic syndrome from International Diabetes Federation; IFG, impaired fasting glucose; AUC, area under the curve (95% confidence interval).

When the analyses were performed separately according to gender, the results were different. In male subjects, an HbA_1c_ of 5.45% predicted the presence of MS with a similar specificity and sensitivity to the whole population (Table 4). However, for IFG an HbA_1c_ of 5.35% gave higher specificity for males. For female subjects, an HbA_1c_ of 5.55% was the appropriate predictive value for both MS criteria, with both specificity and sensitivity > 60%. An HbA_1c_ of 5.45% was the most appropriate cut-off for the prediction of IFG in female subjects (Table 4).

## Discussion

In this large cross-sectional study performed in an Asian population of relatively pure ethnic background an HbA_1c_ of 5.45% was the closest value to the point with ideal sensitivity and specificity for the diagnosis of MS and IFG. Although HbA_1c_ is not considered to be a diagnostic criterion for diabetes or prediabetes, it might provide a simple method of predicting MS or IFG in a large health screening programme. Therefore, the results of this study imply that in non-diabetic Korean subjects with HbA_1c_ > 5.45%, although the subject is not within the diagnostic range for diabetes, life style modification and education for the future development of diabetes and MS should be recommended.

HbA_1c_ is defined as Hb that is irreversibly glycated at one or both N-terminal valines of the β-chains according to the new definition by International Federation of Clinical Chemistry and it does not exclude haemoglobin that is additionally glycated at other sites on the α or β chains [26]. HbA_1c_ could reflect universal tissue protein glycation and might be a much better index of the overall biological effects of glucose above and beyond its predictive value for the 3-month averages of circulating glucose levels [27]. In a nested case–control study of the Women's Health Study cohort [15], high baseline HbA_1c_ predicted future cardiovascular events in women without diabetes mellitus by a factor of 2.25. Osei *et al.*[18] reported that in 219 non-diabetic, obese, first-degree relatives of African-American patients with Type 2 diabetes, the upper tertile of HbA_1c_ reflected some components of MS. These results suggest that HbA_1c_ may be a surrogate marker not only of future diabetes, but also of CVD. Although there are many studies which report the utility of HbA_1c_ in predicting CVD and diabetes, there are few which investigate the usefulness of HbA_1c_ as a predictor of MS.

In our result, an HbA_1c_ of 5.45% was the most appropriate cut-off for MS, defined by both ATP III and IDF guidelines, with the best balance between sensitivity and specificity, at least in this studied population. This level of HbA_1c_ also predicted fasting hyperglycaemia (> 5.6 mmol/l). HbA_1c_ has been considered a very convenient and practical screening tool for high-risk populations for primary diabetes and cardiovascular prevention programmes [28]. Our results could be tested prospectively in long-term studies in other ethnic populations at high risk of developing Type 2 diabetes and MS. Prior mortality studies [12–14] in populations mainly without diabetes mellitus have suggested that the predictive value of HbA_1c_ persists in adjusted analyses. Potential differences in study design that may partly account for these disparities include gender differences and the use of different HbA_1c_ assays. However, the predictive value of HbA_1c_ in these studies was largely attributable to its association with other risk factors.

Another interesting result of our analyses was that the predictive value of HbA_1c_ for MS and IFG was dependent on gender, i.e. in female subjects the HbA_1c_ cut-off with the appropriate specificity and sensitivity was higher than in male subjects. There are few comparable data, but HbA_1c_ might vary by gender, so that cut-offs should be determined separately for men and women. Our study suggests that female Korean subjects are at lower risk of MS than male Korean subjects. However, our results could have arisen from the skewed data of this population, because the baseline level of HbA_1c_ was higher in female subjects. This issue must be clarified in a different population of either the same or different ethnic background.

In this study, HOMA-IR increased with increasing quartile of HbA_1c_. Although insulin resistance is regarded as the aetiological mechanism underpinning MS, direct quantification of insulin sensitivity can be difficult and complex in the general population. Simple measures, such as fasting serum insulin, have been used as a surrogate of insulin resistance in previous epidemiological studies. The present data suggest that HbA_1c_ could be a marker not just for glucose, but also for detection of insulin resistance, such as in MS, as HOMA-IR showed a significant correlation with HbA_1c_ (correlation coefficient = 0.157, *P* < 0.01).

In this study, current smokers and alcohol drinkers had lower HbA_1c_ compared with those who did not smoke or drink. This discrepancy is probably due to the exclusion of subjects with diabetes from the study population, so that smokers and drinkers with high HbA_1c_ might have had diabetes and have been excluded. Furthermore, those who exercised more than three times a week had higher HbA_1c_ compared with subjects who did not exercise regularly. The reason for this discrepancy might be explained by the observation that subjects who exercised regularly had a higher BMI than non-exercisers: those with higher HbA_1c_ also had a higher BMI and might have exercised more deliberately than those subjects with lower HbA_1c_ and lower BMI.

Our study has several limitations. First, it was a cross-sectional study, so that a definite relationship between HbA_1c_ and MS cannot be assumed. Further research in a more diverse ethnic group must be done to clarify the relationship. Second, as this study was performed in non-diabetic subjects, bias might have arisen from the distribution of the metabolic parameters. Third, the non-diabetic reference interval of HbA_1c_ in the method used was 4.4–6.4%. However, these values were not based on data from the same population as the participants of the study, but were provided by the manufacturer. Despite these limitations, this is the first study in this field and included a large study population.

In conclusion, HbA_1c_ may be used as a predictor for fasting hyperglycaemia and MS. An HbA_1c_ of 5.45% seemed to provide the best balance between sensitivity and specificity for detecting MS in this population. Increasing HbA_1c_ was associated with increasing cardiovascular risk factors, so that HbA_1c_ might be predictive of the future development of cardiovascular risk factors. These data support the need for a prospective study examining levels of HbA_1c_ and future cardiovascular risk and the metabolic syndrome.

## Competing interests

None to declare.

## Abbreviations

BMI: body mass index
CV: coefficient of variation
CVD: cardiovascular disease
HDL-C: high-density lipoprotein cholesterol
HOMA-IR: homeostatic model assessment-insulin resistance
IDF: International Diabetes Federation
IFG: impaired fasting glucose
IGT: impaired glucose tolerance
LDL-C: low-density lipoprotein cholesterol
MS: metabolic syndrome
NGSP: National Glycohemoglobin Standardization Program
ROC: receiver–operating characteristic
